# Genome Sequence of the Soybean Cyst Nematode (Heterodera glycines) Endosymbiont “Candidatus Cardinium hertigii” Strain cHgTN10

**DOI:** 10.1128/genomeA.00624-18

**Published:** 2018-06-28

**Authors:** Kurt C. Showmaker, Kimberly K. O. Walden, Christopher J. Fields, Kris N. Lambert, Matthew E. Hudson

**Affiliations:** a Department of Crop Science, University of Illinois at Urbana-Champaign, Urbana, Illinois, USA; b Carl R. Woese Institute for Genomic Biology, University of Illinois at Urbana-Champaign, Urbana, Illinois, USA

## Abstract

In this study, we present the genome sequence of the “Candidatus Cardinium hertigii” strain cHgTN10, an endosymbiotic bacterium of the plant-parasitic nematode Heterodera glycines. This is the first genome assembly reported for an endosymbiont directly sequenced from a tylenchid nematode.

## GENOME ANNOUNCEMENT

The soybean cyst nematode (SCN [Heterodera glycines]) causes more economic loss to soybean (Glycine max) than any other biotic pathogen of this important crop (1). Although an abundance of molecular studies have highlighted the nematode’s interaction with its primary host, soybean, little is known about the molecular interactions between the SCN and its Cardinium endosymbiont. Molecular cloning studies of the 16S RNA and *gyrB* genes within an SCN endosymbiont identified the bacterium as “Candidatus Paenicardinium endonii,” thereafter taxonomically reassigned to “Candidatus Cardinium hertigii” group B (2, 3).

DNA was extracted from surface-sterilized second-stage juveniles of H. glycines population TN10 (Hg type 0) cultured on the susceptible soybean cultivar Essex on soil in the greenhouse. Genomic DNA was extracted using a standard proteinase K protocol (4). PacBio RS II and Illumina TruSeq DNA libraries were prepared from the extracted DNA. The PacBio library was sequenced on 20 single-molecule real-time (SMRT) cells. The Illumina DNA library was sequenced as paired ends (2 × 260) on the Illumina HiSeq 2500 platform. The PacBio reads were assembled with the Canu (version 1.4) long-read assembler (5). The resultant metagenomic assembly was error corrected with Pilon (version 1.22) (6) using the Illumina reads aligned with the BWA-MEM (version 0.7.15) alignment algorithm (7). For the identification of potential endosymbiont contigs within the endosymbiont-nematode metagenome assembly, a BlobTools (version 0.9.19.6) (8) database was built with BWA-MEM alignments to the contigs, and the results from the contigs were aligned to the UniProt uniref100 database with the DIAMOND (version 0.9.10) (9) aligner, using the blastx parameter. Additionally, previously identified 16S RNA (GenBank accession no. DQ314214) and *gyrB* (GenBank accession no. DQ314215) genes for “*Ca*. Cardinium hertigii” were aligned with blastn, and H. glycines RNA sequencing (RNA-seq) reads from NCBI BioProject no. PRJNA415980 (10) were aligned with TopHat (version 2.1.1) (11). All contigs identified as bacterial by BlobTools were manually inspected for the presence of RNA splicing. A single 1.2-Mb contig was identified as the “*Ca*. Cardinium hertigii” chromosome sequence based upon the following 4 supporting criteria: (i) had a predicted circular link to itself, (ii) was taxonomically identified as bacterial, (iii) contained the “*Ca*. Cardinium hertigii” *gyrB* and 16S RNA alignments, and (iv) did not show evidence of RNA splicing. This contig was circularized using Circlator (version 1.5.1) (12) and subsequently error corrected using 2 passes through Quiver and 2 additional passes through Pilon. The resultant 1.2-Mb genome assembly is reported here.

Gene prediction with Prokka (version 1.13) (13) resulted in 973 protein-coding genes and 37 tRNAs. BLASTP alignments of the predicted cHgTN10 proteins to the NCBI NR database (accessed 8 February 2018) resulted in 770 (79%) cHgTN10 predicted proteins with a bit score greater than 60, of which 594 (61%) proteins had a highest scoring blastp alignment to 1 of the 2 previously reported “*Ca*. Cardinium hertigii” strains, cEper1 and cBtQ1 (14, 15). Functional annotation with InterProScan (16) resulted in the identification of several proteins and protein domains similar to those of “*Ca*. Cardinium hertigii,” including the following: Ankyrin repeats, ABC transporters, permeases, a WH2 motif, and DEAD box helicase, as reported for cEper1 (14).

### Accession number(s).

This whole-genome shotgun project has been deposited in DDBJ/ENA/GenBank under the accession no. CP029619. The version described in this paper is the first version, CP029619.1.
